# Association of cigarette and electronic nicotine delivery systems use with internalizing and externalizing problems among US adults: Findings from wave 3 (2015–2016) of the PATH study

**DOI:** 10.1371/journal.pone.0253061

**Published:** 2021-06-15

**Authors:** Bekir Kaplan, Johannes Thrul, Joanna E. Cohen

**Affiliations:** 1 Institute for Global Tobacco Control, Department of Health, Behavior and Society, Johns Hopkins Bloomberg School of Public Health, Baltimore, Maryland, United States of America; 2 Department of Mental Health, Johns Hopkins Bloomberg School of Public Health, Baltimore, Maryland, United States of America; 3 Sidney Kimmel Comprehensive Cancer Center at Johns Hopkins, Baltimore, Maryland, United States of America; University of California San Diego School of Medicine, UNITED STATES

## Abstract

**Aims:**

Electronic Nicotine Delivery System (ENDS) use is increasing among US adults. While existing research has demonstrated higher cigarette smoking rates among people with mental health conditions, there is sparse information on the association between ENDS use and mental health such as internalizing and externalizing problems. The aim of this study was to evaluate associations between internalizing and externalizing problems for cigarette only, ENDS only, and dual users, as well as changes in mental health among those groups.

**Method:**

We used the U.S. Population Assessment of Tobacco and Health (PATH) Study Wave 3 adult data. Internalizing and externalizing problems were self-reported and assessed via the Global Appraisal of Individual Needs-Short Screener; response options were dichotomized to reflect past 12 months and any lifetime problems. Self-reported changes in mental health over the past 12 months were also assessed. Participants’ tobacco use status was categorized as ENDS only use (n = 618), cigarette only use (n = 6,779), dual use (cigarettes and ENDS) (n = 681), and non-current use (n = 16,515).

**Results:**

Lifetime and past 12 month internalizing problems were reported by 63.8% (n = 18,706) and 50.4% (n = 15,326), respectively, while lifetime and past 12 months externalizing problems were reported by 63.3% (n = 18,835) and 52.7% (n = 16,005), respectively. Six percent of participants reported worse mental health over the past 12 months. Compared to non-current use of any tobacco product, and adjusting for age, sex, race, education, and household income, those reporting ENDS use only had higher odds of ever (aOR = 1.52; 95%CI: 1.22–1.89) and past 12 months (aOR = 1.49; 95%CI: 1.22–1.84) internalizing, and externalizing problems (aOR = 1.32; 95%CI: 1.04–1.66 and aOR = 1.34; 95%CI: 1.07–1.67, respectively), and higher odds of reporting worse mental health over the past 12 months (aOR = 1.50; 95%CI: 1.05–2.12).

**Conclusion:**

Health care providers should be aware that internalizing and externalizing problems, and worsening mental health, are more common among adults who use ENDS, cigarettes, or both of these tobacco products.

## Introduction

There has been a substantial increase on the popularity of electronic nicotine delivery systems (ENDS) in the US since they entered the market [1]. During 2017–2019, current ENDS use increased from 2.8% to 4.5% for adults [2, 3]. In 2019, ~10.9 million adults currently used ENDS every day or some days in the US [3].

Approximately 25% of US adults have mental health conditions (MHCs) [4]. There is extensive information that people with MHCs are more likely to smoke cigarettes compared to those without MHCs [5–7]. However, despite the plethora of literature on the association between cigarette smoking and MHCs, relatively few studies have assessed the association between ENDS use and MHCs with various MHC outcomes. A nationally representative study reported higher level of cigarette, ENDS, and dual use among adults with serious psychological distress (SPD) compared to adults without SPD [8]. The association between higher ENDS use and SPD were also reported in several other representative studies [9, 10].

Few studies, however, have investigated mental distress in a more nuanced way, separating out internalizing and externalizing mental health problems. The internalizing problem spectrum consists of depression, anxiety disorders, post-traumatic stress disorders, and eating disorders. The externalizing problem spectrum consists of substance use disorder, antisocial behavior, aggression, and the disruptive behavior disorders (conduct disorder, oppositional defiant disorder, and ADHD) [11]. Using the Behavioral Risk Factor Surveillance System, one nationally representative study [12] reported significant associations between ENDS use and the internalizing disorder depression. To date, only one nationally representative study assessed the association between ENDS use and internalizing and externalizing disorders in adults. Using Population Assessment of Tobacco and Health (PATH) Study Wave 1 data, Conway et al. [13] found that adults using ENDS were more likely to report internalizing and externalizing problems compared to those not using any tobacco products. However, this study did not separate different ENDS use groups, such as exclusive ENDS use or dual use of ENDS and cigarettes.

Given changes in the tobacco product landscape, information on the prevalence of tobacco use and MHCs is needed in order to inform healthcare providers and public health officials. Ultimately, this information may also be used in the development of treatment plans for individuals who are using tobacco products and have MHCs. Thus, the current study aimed to assess associations between internalizing and externalizing problems and ENDS only use, cigarette only use, and dual use of cigarettes and ENDS, using the nationally representative PATH Study Wave 3 (2015–2016) adult data. In addition, we investigated associations between self-reported change on mental health over the past 12 months and ENDS, cigarette, and dual use groups.

## Method

### Data source

This study uses adult data from Wave 3 (October 2015–2016) of the PATH Study [14], a large, nationally representative, cohort study designed to collect information about use of various tobacco products including cigarettes and ENDS, tobacco dependence, cessation, and perceptions of risk and harm, overall physical and mental health, peer and family influences, other substance use, and demographic information [14, 15]. The weighted retention rate between wave 1 and wave 3 was 78.4%. Information on the sampling procedures can be found in the PATH User Guide [14].

### Current smoking status and ENDS use

The current ENDS use and cigarette smoking status of participants was generated using several derived variables that measure tobacco use. Current established cigarette smoking and current established ENDS use were the main two derived variables used. Established cigarette smokers (n = 6,779) were defined as adults who smoked at least 100 cigarettes in their lifetime, and currently smoked every day or some days. Participants in the ENDS use group (n = 618) were adults who currently use ENDS every day or some days. Participants who currently used cigarette and ENDS were classified into a “dual use” group (n = 681). Current cigarette and/or ENDS users who used any other tobacco products were excluded from the analysis (n = 291); further, exclusive users of other tobacco product were excluded. Participants who reported currently not using any tobacco products were classified into a “non-currently use” group (n = 16,515).

### Assessment of mental health

Participants’ mental health status was separately determined for internalizing and externalizing problems. Internalizing (four items) and externalizing problems (seven items) were assessed via the Global Appraisal of Individual Needs—Short Screener (GAIN-SS), modified for the PATH Study (Table 1) [13].

**Table 1:** GAIN-SS subscales and items.

Internalizing Problem Symptoms*	**When was the last time that you had significant problems with …**
	1) Feeling very trapped, lonely, sad, blue, depressed, or hopeless about the future?
	2) Sleep trouble, such as bad dreams, sleeping restlessly, or falling asleep during the day?
	3) Feeling very anxious, nervous, tense, scared, panicked, or like something bad was going to happen?
	4) Becoming very distressed and upset when something reminded you of the past?
Externalizing Problem Symptoms*	**When was the last time that you did any of the following things two or more times …**
	1) Lied or conned to get things you wanted or to avoid having to do something?
	2) Had a hard time paying attention at school, work, or home?
	3) Had a hard time listening to instructions at school, work, or home?
	4) Were a bully or threatened other people?
	5) Started physical fights with other people?
	6) Felt restless or the need to run around or climb on things?
	7) Gave answers before the other person finished asking the question?

*Response options for all questions above were as follows: “0” (never), “1” (over a year ago), “2” (2 to 12 months), and “3” (past month)

Using a continuous measure of severity, the GAIN-SS identifies the individuals at risk for mental health [16]. The response options for all internalizing and externalizing problems were the same and coded as follows: “0” (never), “1” (over a year ago), “2” (2 to 12 months), and “3” (past month). Participants were grouped into three categories based on their responses to the internalizing and externalizing problems: 1) Participants who never reported any internalizing and externalizing problems, 2) Participants who reported at least one problem in the past 12 months, 3) Participants who reported at least one problem more than a year ago but not in the past 12 months (Table 2). For the analyses, the response options were first dichotomized as “0” (never) and “1” (ever) and then “0” (never/more than a year ago) and “1” (past 12 months) for the chi-square and the logistic regression analyses to assess the association between tobacco use and “ever” and “past 12-month” internalizing and externalizing problems, respectively. In addition, self-reported change in mental health status over the past 12 months was assessed with the following question: “Compared with 12 months ago, would you say your mental health is now better, worse, or about the same?” The responses were dichotomized as “0” (better/about the same) and “1” (worse).

**Table 2:** Sociodemographic characteristics of participants.

Characteristics	Groups	n (%)
**Sex**	Male	13,762 (48.0)
	Female	14,301 (52.0)
**Age (Years)**	18–24	8,436 (12.4)
	≥25	19,651 (87.6)
**Race/Ethnicity**	Non-Hispanic White	16,284 (64.4)
	Non-Hispanic Black	4,069 (11.0)
	Hispanic	5,234 (15.2)
	Other	2,538 (9.4)
**Education Level**	Less than High School/GED	5,502 (16.3)
	High School Graduate	6,729 (23.0)
	Beyond High School	15,736 (60.7)
**Income ($)**	<10K	4,623 (11.6)
	10–24.9K	5,680 (19.1)
	25–49.9K	5,973 (22.8)
	50–99.9K	5,863 (26.7)
	≥100K	3,915 (19.8)
**Marital Status**	Married	10,359 (52.5)
	Widowed	4,981 (20.9)
	Never Married	12,371 (26.6)
**Tobacco Use Status**	Cigarettes Only	6,779 (15.2)
	ENDS Only	618 (1.3)
	Dual Use	681 (1.5)
	Nonuse	16,515 (82.0)
**Compared to 12 Months Ago, Mental Health is:**	Better/Same	25,744 (93.8)
	Worse	2,328 (6.2)
**Internalizing Problems**	Never	9,370 (36.2)
	Last Year	15,326 (50.4)
	More Than A Year Ago	3,380 (13.4)
**Externalizing Problems**	Never	9,251 (36.7)
	Last Year	16,005 (52.7)
	More Than A Year Ago	2,830 (10.6)
**Total**		**28,089 (100.0)**

*ENDS*: *Electronic Nicotine Delivery Systems*

### Covariates

The following covariates were used to control for potential confounding: age [18–24; 25+]; sex [male; female]; race/ethnicity [non-Hispanic white; non-Hispanic black; Hispanic; other]; education [less than high school; high school graduate/GED; beyond high school]; household income per year [<$10K, $10–24.9K, $25–49.9K, $50–99.9K, and $≥100K]; marital status [married, widowed, never married] [14].

### Statistical analysis

All analyses were performed using STATA version 15.1 (Statacorp., College Station, TX). The PATH Study population weights were used to adjust for the complex study design including oversampling and non-response. Fay’s method, a variant of balanced repeated replication method, was used to form replicative weights in variance estimation in all analyses. The Fay coefficient was specified at the value of 0.30 as recommended by the PATH Study [14]. The weights produce estimates that are representative of the US non-institutionalized, civilian population ages 18 years and older, adjusting for non-response from Wave 3. We present unweighted counts and weighted percentages in the tables. Further information on the weighting procedure can be obtained from the PATH Study Public-Use Files [14].

Weighted frequency distributions and the chi-square test were used to examine the associations between tobacco use status and internalizing and externalizing problems, as well as the covariates. Logistic regression models were used to assess the association between mental health outcomes and tobacco use groups, controlling for covariates. Since the current study is a secondary data analysis of an existing data, it has exemption report from the Institutional Review Board of the Johns Hopkins Bloomberg School of Public Health.

## Results

The analytic sample of this study included 28,089 adults: 52% were female, 87.6% were aged 25 and over, 64.4% were non-Hispanic white, 60.7% had beyond high school education, 11.6% had less than $10K yearly income, 52.5% were married, 618 (1.3%) currently used ENDS only, 6,779 (15.2%) currently used cigarette only, 681 (1.5%) currently used ENDS and cigarettes (dual use), and 16,515 (82.0%) reported no current use of any tobacco products (Table 2). Worse mental health compared to 12 months ago was reported by 6.2% of respondents (n = 2,328) (Table 2). Lifetime and past 12 months internalizing problems were reported by 63.8% (n = 18,706) and 50.4%(n = 15,326) of respondents, respectively, while lifetime and past 12 months externalizing problems were reported by 63.3% (n = 18,835) and 52.7% (n = 16,005) of respondents, respectively (Table 2).

Lifetime and past 12 months internalizing problems were reported by 73.9% and 61.2% of participants who used ENDS only, 69.1% and 57.4% of participants who used cigarettes only, 76.5% and 65.1% of participants reporting dual use, and 62.5% and 48.5% of participants reporting non-currently using any tobacco products, respectively (p<0.01). After adjustment for sex, age, race/ethnicity, education level, income, and marital status, the adjusted odds ratios (aORs) of reporting ever having internalizing problems were 1.52 (95%CI: 1.22–1.89) for ENDS only use, 1.27 (95%CI: 1.16–1.37) for cigarette only use, and 1.68 (95%CI: 1.32–2.14) for dual use compared to no current use (Table 3). The aORs of reporting past 12 months internalizing problems were 1.49 (95%CI: 1.22–1.84) for ENDS only use, 1.32 (95%CI: 1.23–1.43) for cigarette only use, and 1.67 (95%CI: 1.37–2.04) for dual use compared to no current use (Table 3).

**Table 3:** Association between ever* and past 12 months** internalizing problems and sociodemographic characteristics.

Characteristics	Ever Internalizing Problems			Past 12M Internalizing Problems		
Sex	n	%^a^	aOR (95%CI)	n	%^a^	aOR (95%CI)
Female	8,364	58.8	**1.54 (1.41–1.67)**	8,653	55.0	**1.46 (1.34–1.59)**
Male	10,325	68.5	1	6,660	45.5	1
**Age (Year)**						
18–24	5,595	67.1	1.04 (0.93–1.16)	4,854	57.6	**1.21 (1.10–1.35)**
≥25	13,110	63.4	1	10,471	49.4	1
**Race/Ethnicity**						
Non-Hispanic White	11,256	66.1	1.41 (1.20–1.65)	9,246	52.5	1.32 (1.14–1.55)
Non-Hispanic Black	2,432	59.1	0.96 (0.79–1.16)	1,986	46.5	0.91 (0.75–1.10)
Hispanic	3,297	60.8	1.12 (0.93–1.35)	2,671	47.1	1.00 (0.84–1.19)
Other	1,721	58.5	1	1,423	46.1	1
**Education Level**						
Less than High School/GED	3,551	61.5	**0.79 (0.70–0.90)**	3,002	50.0	**0.86 (0.76–0.97)**
High School Graduate	4,232	60.2	**0.73 (0.64–0.82)**	3,547	48.9	**0.83 (0.64–0.94)**
Beyond High School	10,877	65.9	1	8,739	51.2	1
**Income ($)**						
<10K	3,066	66.8	**1.30 (1.09–1.55)**	2,655	56.9	**1.52 (1.29–1.79)**
10–24.9K	4,009	67.0	**1.29 (1.12–1.49)**	3,353	55.2	**1.43 (1.24–1.64)**
25–49.9K	4,018	65.8	**1.29 (1.11–1.49)**	3,279	51.7	**1.31 (1.15–1.49)**
50–99.9K	3,953	65.3	**1.26 (1.10–1.44)**	3,141	50.2	**1.23 (1.06–1.43)**
≥100K	2,478	60.1	1	1,909	44.6	1
**Marital Status**						
Widowed	3,554	70.0	**1.43 (1.30–1.59)**	5,021	56.8	**1.34 (1.22–1.48)**
Never Married	8,458	68.1	**1.37 (1.25–1.51)**	2,920	56.3	**1.33 (1.21–1.45)**
Married	6,466	59.5	1	7,195	45.1	1
**Tobacco Use Status**						
ENDS Only	450	73.9	**1.52 (1.22–1.89)**	377	61.2	**1.49 (1.22–1.84)**
Cigarettes Only	4,706	69.1	**1.27 (1.16–1.37)**	3,935	57.4	**1.32 (1.23–1.43)**
Dual Use	519	76.5	**1.68 (1.32–2.14)**	447	65.1	**1.67 (1.37–2.04)**
Nonuse	10,763	62.5	1	13,427	48.5	1

*Compared to participants who reported “never” having internalizing problems.

**Compared to participants who reported internalizing problems “never” or “more than a year ago”

a) All chi-square p-values were <0.001 except for education and past 12 months internalizing problems where p = 0.153.

Reporting ever and past 12 months externalizing problems was 71.6% and 62.0% for ENDS only use, 65.0% and 54.1% for cigarette only use, 73.9% and 61.8% for dual use, and 62.3% and 51.8% for not currently using any tobacco products, respectively (p<0.01). After adjustment for sex, age, race/ethnicity, education level, income, and marital status, the aORs of reporting ever externalizing problems were 1.32 (95%CI: 1.04–1.66) for ENDS only use, 1.23 (95%CI: 1.13–1.34) for cigarette only use, and 1.55 (95%CI: 1.23–1.96) for dual use compared to no current use (Table 4). The aORs of reporting past 12 months externalizing problems were 1.34 (95%CI: 1.07–1.67) for ENDS only use, 1.23 (95%CI: 1.14–1.32) for cigarette only use, and 1.37 (95%CI: 1.13–1.67) for dual use compared to no current use (Table 4).

**Table 4:** Association between ever* and past 12 months** externalizing problems and sociodemographic characteristics.

Characteristics	Ever Externalizing Problems			Past 12M Externalizing Problems		
Sex	n	% ^a^	aOR (95%CI)	n	% ^a^	aOR (95%CI)
Female	9,814	63.7	**1.08 (1.00–1.16)**	8,515	54.0	**1.18 (1.10–1.28)**
Male	9,006	62.8	1	7,478	51.2	1
**Age (Year)**						
18–24	5,926	71.1	**1.37 (1.22–1.55)**	5,264	63.0	**1.50 (1.35–1.67)**
≥25	12,908	62.2	1	10,740	51.2	1
**Race/Ethnicity**						
Non-Hispanic White	11,663	68.3	**1.62 (1.32–1.99)**	9,992	57.6	**1.49 (1.22–1.83)**
Non-Hispanic Black	2,328	55.4	0.95 (0.78–1.18)	1,934	43.7	0.85 (0.70–1.05)
Hispanic	3,103	51.4	0.95 (0.77–1.17)	2,582	40.5	0.84 (0.68–1.04)
Other	1,741	57.5	1	1,497	48.7	1
**Education Level**						
Less than High School/GED	3,265	52.3	**0.60 (0.54–0.69)**	2,697	41.6	**0.62 (0.55–0.70)**
High School Graduate	4,082	55.2	**0.60 (0.54–0.68)**	3,420	45.1	**0.64 (0.57–0.73)**
Beyond High School	11,444	69.4	1	9,849	58.6	1
**Income ($)**						
<10K	2,867	57.6	**0.71 (0.59–0.86)**	2,429	47.8	**0.73 (0.62–0.87)**
10–24.9K	3,731	59.2	**0.75 (0.64–0.88)**	3,152	48.8	**0.76 (0.67–0.86)**
25–49.9K	4,055	64.4	0.90 (0.78–1.03)	3,417	52.7	**0.84 (0.74–0.96)**
50–99.9K	4,175	66.9	0.91 (0.78–1.05)	3,516	55.3	**0.85 (0.75–0.97)**
≥100K	2,852	70.3	1	2,492	60.4	1
**Marital Status**						
Widowed	3,259	62.4	**1.18 (1.04–1.34)**	5,484	50.7	**1.09 (0.97–1.22)**
Never Married	8,704	68.8	**1.43 (1.26–1.63)**	2,715	59.1	**1.39 (1.23–1.58)**
Married	6,632	61.2	1	7,603	50.4	1
**Tobacco Use Status**						
ENDS Only	438	71.6	**1.32 (1.04–1.66)**	385	62.0	**1.34 (1.07–1.67)**
Cigarettes Only	4,484	65.0	**1.23 (1.13–1.34)**	3,759	54.1	**1.23 (1.14–1.32)**
Dual Use	501	73.9	**1.55 (1.23–1.96)**	425	61.8	**1.37 (1.13–1.67)**
Nonuse	11,029	62.3	1	9,408	51.8	1

*Compared to participants who reported “never” having externalizing problems.

**Compared to participants who reported externalizing problems “never” or “more than a year ago”

a) All chi-square p-values were <0.001 except for gender and ever externalizing problems where p = 0.270.

Reporting lifetime or past 12 months internalizing and externalizing problems between participants who use ENDS only, cigarettes only, and both cigarettes and ENDS was significantly different in univariate analyses (p<0.05), however, these differences did not remain significant in adjusted regression analyses (p>0.05).

We conducted a sensitivity analysis to assess the association between ENDS use and mental health condition among exclusive ENDS users who are not former cigarette smokers and ENDS users who are former smokers. After adjustment for sex, age, race/ethnicity, education level, income, and marital status, the adjusted odds ratios (aORs) of reporting ever and past 12 months internalizing problems were 1.31 (95%CI: 0.85–2.14) and 1.30 (95%CI: 0.87–1.92) for exclusive ENDS users who are not former cigarette smokers (S1 Table). The adjusted ORs for reporting ever and past 12 months externalizing problems were 1.09 (95%CI: 0.68–1.75) and 1.16 (95%CI: 0.75–1.81) for exclusive ENDS users who are not former cigarette smokers (S2 Table). The ORs for ENDS users who are former smokers were almost identical to those for ENDS only users (S1 and S2 Tables).

Overall, the percentage of participants reporting worse mental health over the past 12 months was 6.2% (n = 2,328). Reporting worse mental health compared to 12 months ago was reported by 8.7% respondents for ENDS only use, 9.6% respondents for cigarette only use, 11.4% of respondents for dual use, and 5.9% respondents for no current tobacco use (p<0.01). After adjustment for sex, age, race/ethnicity, education level, income, and marital status, the aORs of reporting worse mental health were 1.50 (95%CI: 1.05–2.12) for ENDS only use, 1.83 (95%CI: 1.56–2.16) for cigarette only use, and 2.02 (95%CI: 1.50–2.73) for dual use compared to no current use (Table 5). Reporting worse mental health was not significant between participants who use ENDS only, cigarette only, and both cigarettes and ENDS in the adjusted logistic regression model (p>0.05).

**Table 5:** Association between reporting worse mental health compared to 12 months ago and sociodemographic characteristics.

Characteristics	Worse Mental Health		
Sex	n	%	aOR (95%CI)
Female	1,364	7.0	**1.37 (1.19–1.58)**
Male	964	5.3	1
**Age (Year)**			
18–24	894	10.2	**1.82 (1.55–2.14)**
≥25	1434	5.6	1
**Race/Ethnicity**			
Non-Hispanic White	1428	6.6	**1.40 (1.11–1.77)**
Non-Hispanic Black	298	5.4	0.87 (0.66–1.15)
Hispanic	384	5.4	0.99 (0.75–1.33)
Other	218	5.3	1
**Education Level**			
Less than High School/GED	504	6.9	**0.75 (0.60–0.94)**
High School Graduate	534	5.3	**0.66 (0.54–0.82)**
Beyond High School	1,275	6.3	1
**Income ($)**			
<10K	548	10.0	**2.05 (1.56–2.69)**
10–24.9K	528	7.3	**1.47 (1.13–1.92)**
25–49.9K	471	6.1	1.28 (1.00–1.65)
50–99.9K	371	5.1	1.07 (0.84–1.38)
≥100K	254	4.7	1
**Marital Status**			
Widowed	497	7.7	1.23 (0.98–1.55)
Never Married	1,178	7.9	1.12 (0.94–1.34)
Married	617	4.7	1
**Tobacco Use Status**			
ENDS Only	54	8.7	**1.50 (1.05–2.12)**
Cigarettes Only	701	9.6	**1.83 (1.56–2.16)**
Dual Use	83	11.4	**2.02 (1.50–2.73)**
Nonuse	1,113	5.1	1

*All chi-square p-values were less than 0.05.

## Discussion

This cross-sectional nationally representative study of adults in the US found that individuals who use ENDS only, cigarettes only, and cigarettes and ENDS were more likely to report ever and past 12 months internalizing and externalizing problems, compared to respondents reporting no current use of tobacco products. In addition, reporting worse mental health compared to 12 months ago was higher among participants who used ENDS only, cigarettes only, and cigarettes and ENDS compared to those who did not currently use any tobacco product.

The results of this study are consistent with the current literature. A recent study using PATH Study Wave 1 data (2013–2014) reported that individuals reporting current tobacco use including ENDS use had higher levels of internalizing and externalizing problems compared to those reporting no tobacco use [13]. The current study, however, expands these previous findings by demonstrating that respondents reporting current ENDS only use and dual use of ENDS and cigarettes also reported higher levels of these problems. Another recent nationally representative study reported that people who use ENDS were more likely to report depression compared to those who did not currently use any tobacco product [12]. Our current study found that this association extends to internalizing problems more broadly, which could have implications for public health and clinical practice. While the associations between cigarette use and MHCs is well-known [4], results of current study add to the literature on the association between ENDS use and MHCs. It is critical for health care providers to screen patients for ENDS use, especially those who are diagnosed with internalizing and externalizing problems.

In this study, self-reported worse mental health compared to 12 months ago was higher among adults who used ENDS only, cigarettes only, and cigarettes and ENDS, which is consistent with existing literature. For example, a longitudinal study of adolescents reported that, compared to youth reporting no tobacco use, depression symptoms over a 12-month follow-up period increased more among youth reporting ENDS only use [17]. In another longitudinal study, greater odds of depression and anxiety were also reported among youth reporting polytobacco use compared to youth who did not use any tobacco product [18]. In addition, previous research established that long-time nicotine exposure may negatively affect the brain by disrupting dopamine pathways [19], amplifying stress sensitivity [20], and dysregulating neural pathways underlying emotional processing [21]. Taking these findings into account and interpreting them together with findings of our study, it can be argued that the worsening of mental health conditions is likely not just a problem among individuals who smoke cigarettes or report dual use of cigarettes and ENDS, but may also be an issue among those who use ENDS only compared to those who do not currently use any tobacco product. Therefore, further research on the causal association between ENDS use and MHCs and potential contribution of ENDS to worsening mental health is warranted.

This study has several limitations. First, the association between MHCs and tobacco use is cross-sectional. Therefore, our study does not address the causal association between MHCs and use of different tobacco products among adults. Second, internalizing and externalizing problems relied on self-reports and do not represent a clinical diagnosis of a mental disorder. Third, tobacco use status of participants was based on self-reported information and did not include biological verification of tobacco product use. Fourth, the association between mental health condition and exclusive ENDS user who are not former cigarette smokers was not statistically significant due to the small sample size; these findings seem mostly driven by ENDS users who are former smokers. The findings for exclusive ENDS users who are not former cigarette smokers are inconclusive in this sample and need to be replicated in future research with larger sample size. Despite these limitations, relying on nationally representative data is a strength of this study.

## Conclusion

Internalizing and externalizing problems are more common among those who currently use ENDS only, cigarettes only, and cigarettes and ENDS (dual use), compared to those not currently using tobacco products. In addition, reporting worse mental health compared to the last year was higher among these different tobacco product use groups, compared to the non-use group. Further studies are needed to expand on our cross-sectional analysis and determine directionality and causality of this association. Health care providers should be aware that worsening mental health, internalizing, and externalizing problems, are more common among adults who use ENDS, cigarettes, or both of these tobacco products. Findings of the current study can inform screening and treatment of tobacco product use as well as interventions focusing on comorbidities between tobacco use and mental disorders.

## Supporting information

S1 Table Association between ever* and past 12 months** internalizing problems and sociodemographic characteristics. (DOCX)

S2 Table Association between ever* and past 12 months** externalizing problems and sociodemographic characteristics. (DOCX)
