# Supplementary material for: Association of cigarette and electronic nicotine delivery systems use with internalizing and externalizing problems among US adults: Findings from wave 3 (2015–2016) of the PATH study
Source: PLoS One. 2021 Jun 15;16(6):e0253061. doi: 10.1371/journal.pone.0253061 (PMC8205124; doi:10.1371/journal.pone.0253061)
Supplement: S1 Table — (DOCX) [file pone.0253061.s001.docx]

| **S1 Table: Association between ever* and past 12 months** internalizing problems and sociodemographic characteristics** | | | | | | |
| --- | --- | --- | --- | --- | --- | --- |
| **Characteristics** | **Ever Internalizing Problems** | | | **Past 12M Internalizing Problems** | | |
| **Tobacco Use Status** | n | % | aOR (95%CI) | n | % | aOR (95%CI) |
| Dual Use | 519 | 76.5 | **1.68 (1.32-2.14)** | 447 | 65.1 | **1.67 (1.37-2.04)** |
| Cigarettes Only | 4,706 | 69.1 | **1.27 (1.16-1.37)** | 3,935 | 57.4 | **1.32 (1.23-1.43)** |
| All ENDS Only (n=618) | 450 | 73.9 | **1.52 (1.22-1.89)** | 377 | 61.2 | **1.49 (1.22-1.84)** |
| ENDS only users who are not former cigarette users (n=193) | 135 | 71.5 | 1.31 (0.85-2.14) | 75 | 38.9 | 1.30 (0.87-1.92) |
| ENDS only users who are former cigarette users (n=425) | 323 | 73.6 | **1.51 (1.20-1.89)** | 172 | 39.5 | **1.51 (1.20-1.90)** |
| Nonuse | 10,763 | 62.5 | 1 | 13,427 | 48.5 | 1 |
| *Compared to participants who reported “never” having internalizing problems.  **Compared to participants who reported internalizing problems “never” or “more than a year ago.” | | | | | | |
