# Supplementary material for: Association of cigarette and electronic nicotine delivery systems use with internalizing and externalizing problems among US adults: Findings from wave 3 (2015–2016) of the PATH study
Source: PLoS One. 2021 Jun 15;16(6):e0253061. doi: 10.1371/journal.pone.0253061 (PMC8205124; doi:10.1371/journal.pone.0253061)
Supplement: S2 Table — (DOCX) [file pone.0253061.s002.docx]

| **S2 Table: Association between ever* and past 12 months** externalizing problems and sociodemographic characteristics** | | | | | | |
| --- | --- | --- | --- | --- | --- | --- |
| **Characteristics** | **Ever Externalizing Problems** | | | **Past 12M Externalizing Problems** | | |
| **Tobacco Use Status** | n | % | aOR (95%CI) | n | % | aOR (95%CI) |
| Dual Use | 501 | 73.9 | **1.55 (1.23-1.96)** | 425 | 61.8 | **1.37 (1.13-1.67)** |
| Cigarettes Only | 4,484 | 65.0 | **1.23 (1.13-1.34)** | 3,759 | 54.1 | **1.23 (1.14-1.32)** |
| ENDS Only (n=618) | 438 | 71.6 | **1.32 (1.04-1.66)** | 385 | 62.0 | **1.34 (1.07-1.67)** |
| ENDS only users who are not former cigarette users (n=193) | 132 | 70.0 | 1.09 (0.68-1.75) | 123 | 62.8 | 1.16 (0.75-1.81) |
| ENDS only users who are former cigarette users (n=425) | 316 | 71.6 | **1.34 (1.03-1.75)** | 271 | 61.3 | **1.36 (1.06-1.74)** |
| Nonuse | 11,029 | 62.3 | 1 | 9,408 | 51.8 | 1 |
| *Compared to participants who reported “never” having externalizing problems.  **Compared to participants who reported externalizing problems “never” or “more than a year ago.” | | | | | | |
